# The Drivers of Low Vaccination Utilization in Niger

**DOI:** 10.4269/ajtmh.23-0708

**Published:** 2024-02-13

**Authors:** Bernard Seytre, Sanoussi Chaibou, Bernard Simon

**Affiliations:** ^1^ bnscommunication, Klikamé, Lomé, Togo;; ^2^ Faculté des Sciences Infirmières Université Laval, Québec, Canada;; ^3^ Croix-Rouge Française, Paris, France

## Abstract

Vaccination adherence involves the expected benefit of a vaccine and the perceived risk of the disease. To develop an evidence-based communication strategy aimed at improving vaccination coverage in Niger, we conducted a mixed socio-anthropological study of the perceptions among the population on the benefit and the risk of the childhood (Expanded Program on Immunization) and the COVID-19 vaccines. Our results show that negative rumors are not a significant driver of vaccine refusal. The insufficient level of fully vaccinated, compared with partially vaccinated, children might be explained by misunderstandings around the side effects of vaccines and the necessity for full vaccination. Approximately one-fourth of the population is vaccinated against COVID-19, whereas 73.3% think that vaccines against the disease are a “good thing,” and 83% of those who have heard messages promoting the vaccination approve of them. This apparent contradiction is explained by a low perception of the risks of COVID-19. More than half of the population surveyed believe that the disease is not present in the country. A large majority believe that only ill people can transmit the disease, whereas only 12.8% think they know anybody who has ever been sick with COVID-19. Three-fourths of the interviewees have seen images from around the world of persons sick or deceased from COVID-19; the same proportion has not seen any such images of affected patients in Niger. Communication to improve COVID-19 uptake should focus on the reality of the disease presence and its transmission and not on rumors surrounding the vaccines.

## INTRODUCTION

Vaccination coverage in sub-Saharan African countries is inconsistent, with generally high coverage of routine child vaccines included in the Expanded Program on Immunization (EPI) on one hand and low coverage of the COVID-19 vaccines and the Ebola or cholera vaccines used during outbreaks on the other.^1^ However, whereas more than 90% of children receive at least one dose of a vaccine for childhood illnesses, only 56.5% are fully vaccinated, defined as having received all of the required doses of vaccines against six diseases during six vaccination sessions.^2^

In Niger, the percentages of children in 2021 who received the first dose of the pentavalent (diphtheria, tetanus, pertussis, hepatitis B, *Haemophilus influenzae* type B), pneumococcus, and rotavirus vaccines were 81%, 83%, and 80%, respectively.^3^ Only 33% of children were fully vaccinated. Vaccination coverage for COVID-19 attained 22% of the eligible population at the end of 2022, although COVID-19 vaccination was mandatory to cross any border. As part of a Red Cross program to improve the sustainability of EPI and COVID-19 vaccination in Niger, Guinea, and the Comoros (AFROVAX), we conducted a mixed socio-anthropological study on the drivers of vaccination adherence or nonadherence to gather the evidence on which to base a communication strategy.

The decision to get vaccinated depends on an individual’s analysis of the balance between the benefits and risks of the vaccination for their children or for themselves. The benefit of vaccination is protection from the disease, whereas the risk encompasses the vaccine’s potential side effects. These side effects may be perceived as acceptable if a high benefit is expected or as unacceptable if a low benefit is expected.

According to Arce et al., COVID-19 vaccine acceptance in low- and middle-income countries might primarily be explained by an interest in personal protection against the disease, and concern about side effects could be the most common reason for hesitancy.^4^ Studies have evaluated the perception of COVID-19 vaccines and of COVID-19 risk, asking people if they were afraid of COVID-19 or felt at risk for the disease.^5^^,^^6^ However, these studies provide limited information from a communication perspective, for which it is necessary to understand why people trust or distrust the vaccines and why they do or do not feel at risk for the disease.

For the EPI vaccines, we needed to determine why a substantial proportion of parents bring their children to the first vaccination sessions yet do not present at the following ones.

## MATERIALS AND METHODS

From March 10 through March 27, 2023, we conducted a quantitative and a qualitative study in Niamey, the capital and main city of Niger, and in two villages in the Zinder region, Gaffati and Tirmini (Table 1). The investigators received training for 2 days in Niamey before the study.

**Table 1:** Distribution by sex and locality

Locality	*n*
Gaffati	54
Women	36
Men	18
Niamey	400
Women	220
Men	180
Tirmini	55
Women	30
Men	25
Total	509

For the quantitative study, we conducted face-to-face interviews with 509 people, divided into three age groups (18–24 years, 25–59 years, and 60 years and over), according to the national demographics (Table 2). The population size of Niger is approximately 25 million. For a 95% CI, the margin of error for our study is 4%. The questionnaire was conducted in French, and the investigators translated the questions into Hausa or Songhai when necessary. The answers were recorded on tablets and the completed questionnaires were uploaded daily to the Open Data Kit (ODK) platform. The results were exported as Excel files.

**Table 2:** Distribution by age

Age, in years	*n*
18–24	130
25–59	326
60+	52
Total	509

To minimize bias in certain answers that could be induced by the previous questions, we organized the questionnaire to begin with general questions on the topics and end with the questions specific to the COVID-19 vaccines in the following order: causes of infectious diseases, role of vaccines, child vaccination, COVID-19 pandemic, and COVID-19 vaccines. We particularly wanted to avoid a scenario in which a negative opinion of COVID-19 vaccines might influence the opinions on vaccines in general and on COVID-19. Along the same line, we asked general questions on vaccine appreciation before presenting specific questions on the real or presumed side effects. In several instances, we also asked two closely related questions, to confirm the answers.

For the qualitative study, we conducted 10 individual in-depth interviews and four focus groups (Table 3).

**Table 3:** Focus groups and individual interviews

Location	*n*	*n*	Total
Gaffati	2	1	11
Tirmini	2	1	11
Niamey	6	2	18
Total	10	4	40

## RESULTS

### General knowledge and EPI vaccine perceptions.

Knowledge of infectious agents and their role in diseases is very high. Among the respondents, 90.6% answered “yes” to the question, “Do you know what a virus or a microbe is?” When asked “What can a microbe or a virus do when it infects someone?,” 98.7% answered “a disease” among the other possible answers of “a good thing,” “nothing,” or “no answer.”

Among the respondents, 96.9% thought that vaccinating a “young child” is a “good thing”; 97.1% thought that the vaccines “protect against diseases,” which is the most precise answer; and 20.8% thought that they “help them grow,” which could also be considered true (Table 4).

**Table 4:** What is the goal of vaccinating young children? (several possible answers)

Goal	Percent
Protect against diseases	97.1
Help them gain weight	7.7
Help them grow	20.8
Do not know	2.8

We also evaluated, through a series of questions, the impact of the lasting opposition to the oral polio vaccine (OPV) in the neighboring northern Nigerian states, where a rumor circulates claiming that the vaccine sterilizes girls.^7^ To the first question: “Do you think that the oral polio vaccine is a good thing?,” 88.4% of respondents answered “yes” and 9.8% answered “no.” We then asked the following questions: “Have you heard that certain vaccines sterilize girls?” followed by “Do you think certain vaccines sterilize girls?” to which 48.1% stated they had heard the rumor and 12.2% thought it was true.

We evaluated the sources of information on vaccination and the level of trust in these various sources. The two most important sources of information—the community (neighbors and family) and health workers—are almost equal, each having a response rate of approximately 61%. Yet the community is the least trusted among all the proposed sources (57.4% confident, 40.9% suspicious), whereas health workers are the most trusted (95.1% confident, 4.0% suspicious) (Table 5).

**Table 5:** How much do you trust sources of information on vaccines?

Source	How do you get information on vaccines?	Very confident	Moderately confident	A little suspicious	Very suspicious	No answer
Health workers	61.9%	81.7%	13.4%	2.0%	2.0%	1.0%
Neighbors, family	60.7%	24.8%	32.6%	22.2%	18.7%	1.8%
Radio	43.6%	49.9%	29.9%	11.8%	4.9%	3.5%
Traditional leaders	38.7%	46.4%	36.0%	8.4%	3.9%	5.3%
TV	36.5%	48.1%	31.8%	11.0%	3.7%	5.3%
Religious leaders	16.9%	39.1%	36.7%	12.0%	5.5%	6.7%

Among the respondents, 53.2% considered it “very easy” to get children vaccinated and 34.6% considered it “easy.” When asked, “What is most annoying about getting your children vaccinated?,” 69.2% answered, “Nothing, it is easy,” and 21.4% cited the lack of friendliness of the health workers, 16.9% noted the waiting time, and 7.1% cited the distance to the vaccine center.

### COVID-19.

The qualitative study revealed that people do not doubt that COVID-19 exists globally, but that, based on their own personal experience, they believe either 1) COVID-19 is not present in Niger or 2) it does not pose a serious threat.

Following are significant excerpts from the interviews or focus groups.

#### COVID-19 in the world.

“Yes, it definitely exists, but it has not been very harsh here. Anyone who gets information via social networks, TV, or radio knows very well that it exists because it is talked about everywhere.” (Interview with a man, Niamey)

“Of course it is a disease that existed, moreover it is the only disease in the history of humanity that prevented Muslims from making the pilgrimage to Mecca, and in some countries even daily group prayers were forbidden. So it is a disease that everyone has experienced, including Muslim countries, therefore there is no reason to say that it did not exist.” (Interview with a man, Niamey)

“I personally cannot confirm that this is a disease that exists because everything I know about this disease is based on information. I have never been an eyewitness.” (Interview with a man, Niamey)

#### COVID-19 in Niger.

“Yes, there are rumors about its veracity saying that even the authorities who have claimed its existence are not at all convinced. [Laughter] Even the leaders who confirmed it do not believe. Whoever dares to say that it exists will be considered a liar. Or be considered a Christian. And everywhere it is the same rumors because nobody believes in its existence. For example, out of a population of 100 people, it is only the ten percent who believe in this disease.” (Woman from a focus group, Gaffati)

“I do not believe there is a case of COVID-19 in Niger. Even before the advent of the coronavirus we had in Niger a kind of coronavirus, it is the common cold. But it hurts us less than it does Westerners.” (Interview with a man, Niamey)

“Our coronavirus in Niger is the common cold and it only harms clear complexions. When cold touches a Tuareg, it must be isolated from the rest of the population, the cold kills the Tuareg. He can’t do anything against us. Our only coronavirus in Niger is poverty and lack of solidarity.” (Woman from a focus group, Niamey)

“Yes, the coronavirus has affected the whole world, but still here, at home, people were just developing false information. I think Covid-19 never existed in Niger.” (Interview with a man, Niamey)

The quantitative study allowed us to measure the prevalence of these ideas.

As described earlier, we initially asked a series of questions about the COVID-19 pandemic, without mentioning the vaccination. A very large majority (82.9%) thought that COVID-19 exists in the world (6% did not answer), yet less than half (43.8%) thought that it was present in Niger (11.9% did not answer). In response to the question, “Do you think that you could become ill one day with COVID-19?,” 44.2% answered “yes” and 48.1% responded “no”; these answers are consistent with those regarding the presence of COVID-19 in Niger.

We then posed a series of questions to understand what the previous answers were based on. We asked, “Have you seen images on television or social networks of people in the world sick with or dead from COVID-19?” and “Have you seen images on television or social networks of people sick with or dead from COVID-19 in Niger?” 72.5% reported having seen such images in the world and 26.9% had not; 27.1% had seen such images in Niger and 71.5% had not.

The respondents had a good knowledge of the cause of COVID-19: 98.2% had heard of the coronavirus and 74.3% knew that a virus causes COVID-19. However, the transmission of this virus is very poorly understood. To the question “In whom can we find the coronavirus?,” 3.9% answered “everybody” and 87.6% answered “only sick people.” The answers to the following question confirmed this perception. To the question “Who can transmit the COVID-19 disease to you?,” 76.6% answered “a sick person” and 2.0% answered “a person who is not sick,” whereas 7.7% chose “a wild animal,” 3.3% said “a dog or a cat,” 2.2% chose “food,” and 2.0% chose “a farm animal.”

#### COVID-19 vaccines.

We first asked the very general question: “Do you think that COVID-19 vaccines are a good thing?,” to which 73.3% answered “yes” and 21.2% replied “no.” We asked the 108 people who had answered “no” about their response, with five possible answers (Table 6). The first two answers expressed a negative opinion about the vaccines, the third was neutral, and the last two concerned the belief that COVID-19 does not pose a threat. Among the respondents, 45.4% chose one of the two negative answers, and 16.7% chose the other, representing 9.6% and 3.5% of the entire panel, respectively.

**Table 6:** Reasons for the COVID-19 vaccines not to be a good thing

Why are these vaccines not a good thing?	Percentage of the 108 people who were asked this question	Percentage of the whole panel
They cause diseases or deaths	45.4	9.6
They can transmit COVID-19	16.7	3.5
They have no efficacy against this disease	13.0	2.8
They are unnecessary around the world because COVID-19 does not exist	17.6	3.7
They are unnecessary here, as COVID-19 does not exist in Niger	46.3	9.8
No answer	2.8	0.0
Respondents	108	509

We then asked a double question: “Have you heard messages promoting the COVID-19 vaccination?” followed by “Do you support these messages?” for those who had heard the messages. Among the respondents, 82.9% had heard the messages promoting the COVID-19 vaccination, of whom 83% supported those messages, representing 69.2% of the entire panel.

**Figure 1.**
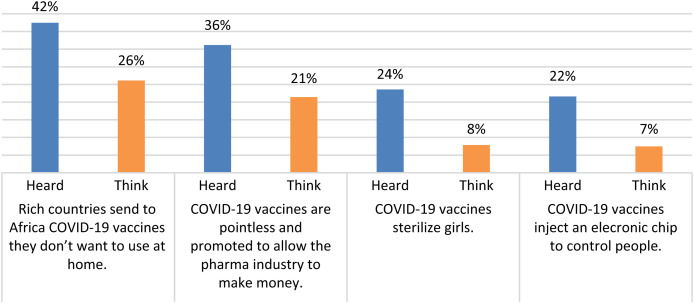
Have you heard … ? Do you think … ? Yes answers. Percentages calculated on the 509 interviewed people.

Finally, we evaluated the impact of four rumors we identified as circulating in Niger, asking again double questions: “Have you heard [*rumor*]?,” followed by “Do you believe [*rumor*]?” (Figure 1). The two rumors related to a purported agenda on the part of the vaccine providers are believed by approximately one-fourth of the population surveyed, and the two rumors that consider the vaccines to be harmful are only believed by 8% and 7%, respectively.

## DISCUSSION

Our study provides some explanations for the drivers of the insufficient adherence to vaccination, especially the COVID-19 vaccination.

Our results show a very high level of awareness among the population of microbes and their role in infectious diseases: 90.6% of those surveyed “know what a virus or a microbe is” and 98.7% know that they cause diseases. This is an asset for communication. Given this knowledge, people can understand explanations that certain preventive measures, such as handwashing, mask wearing, and vaccination, can prevent microbes from spreading diseases.

Our results confirm previous studies conducted in sub-Saharan Africa that showed a high acceptance of child vaccination.^8^ Among our panel members, 96.9% thought that vaccinating their children is a “good thing,” and 97.1% understand that vaccines protect against diseases. However, we found that the appreciation of the OPV as a “good thing” is substantially lower, with 88.4% positive answers, which confirms a specific distrust of this vaccine induced in certain African regions by the succession of national immunization days campaigns conducted by the Global Poliomyelitis Eradication Initiative.^7^

Our study provides clues to the causes for the different percentages of partly and fully vaccinated children. Among the respondents, 30.6% of the panel think that the vaccines have side effects, 21.4% consider the lack of friendliness of the health workers annoying, and 16.9% cite the waiting time as a disincentive. We can hypothesize that health workers do not provide sufficient information about potential side effects of vaccination and the importance of getting a child fully vaccinated. The vaccination sessions could also be better organized to reduce the waiting time. However, the full scope of reasons for dropping out before complete vaccination is achieved, which presents a major public health issue, should be further studied.

Our study provides evidence that the low vaccination rate against COVID-19 is not due to a negative perception of the vaccines, but rather to a major underestimation of the toll of the epidemic in the country.

The most commonly believed rumor of the four we evaluated (26%) is that rich countries send the COVID-19 vaccines that they do not use at home to Africa, a perception that might originate in the fact that when the RNA vaccines became available, some Northern Hemisphere countries donated the AstraZeneca vaccines they were no longer using to the Southern countries. The second rumor (21%) is that the vaccines are useless and are promoted solely for the interest of the pharmaceutical industry, which is directly linked to the belief that COVID-19 is not present in the country. These two rumors do not cast a serious doubt on the efficacy and safety of the vaccines and are probably not sufficient to prevent people from being vaccinated. The two rumors that express a real fear about the vaccines (sterilization of girls and injection of a digital chip) were only believed by 8% and 7% of the panel, respectively.

The differences in percentages we identified on several instances between those people who heard and believed a rumor reveal that people do apply critical thinking to what they hear. This is further confirmed by our results, which show that although the community is one of the main sources of information on vaccines (approximately 61%, equal to the health workers), it is also the least trusted of the six information sources.

The opinion on the COVID-19 vaccines is remarkably, and perhaps surprisingly, high in a country where less than one-fourth of the population is vaccinated, including a substantial proportion who required vaccination to travel abroad. Indeed, 73.3% of the people surveyed think that the COVID-19 vaccines are a “good thing,” and 83% of the people who heard the messages promoting the vaccination support them.

The reason for the low adherence to the COVID-19 vaccination clearly stems from a feeling of not being at risk for the disease, as has been found in various qualitative studies.^6^ Whereas 82.9% of the population understand that the COVID-19 disease exists in the world, only 43.8% know that it is present in Niger, meaning that more than half the population thinks it is not necessary to get vaccinated. Those who do think that the disease is present in the country have a very low perception of their risk of becoming ill: 87.6% think that only sick people can carry and transmit the coronavirus, only 12.8% report that they know someone who has had COVID-19, and only 5.5% know of someone in their own family. We do not know how many of the 12.8% respondents have met with a sick person and are likely to think that the encounter put them at risk. Therefore, the proportion of people who think that they were at risk of getting COVID-19 at some point is between only 5.5% and 12.8%.

If, during the first 2 years of the pandemic, it was widely thought that it did not severely impact Africa, in 2022 studies began to unveil another reality. It is estimated that by September 2021, 65.1% of the African population had been infected at least once by SARS-Cov2, the COVID-19 virus, a proportion that is probably higher today.^9^ In Niger, although 7,371 infections were reported by the end of 2021, the estimated number of infections is 10,874,074.^10^ For 2020–2021, 275 COVID-19 deaths were reported in Niger; however, an estimate of the direct COVID-19 deaths for the same period is 14,222, and two reported estimates of the excess deaths (direct and due to disruptions in health care) are 9,622 and 18,100.^11^^,^^12^ This major underestimation of the epidemic concerns all of sub-Saharan Africa, where it is estimated that only 1.4% of the SARS-Cov2 infections, 35.3% of the deaths, and 7% of the excessed deaths were reported.

Given the severely underestimated burden of the disease, it is not surprising that so few people think that they know someone who has had COVID-19. In addition, this personal experience has been reinforced by the news reports: 72.5% of the people we interviewed reported having seen images from around the world of people sick or dying from COVID-19, 71.5% had not seen any such images from Niger. A previous qualitative study we conducted in five West African countries in 2021 yielded similar results.^13^

Various studies conducted in sub-Saharan Africa have concluded vaccine hesitancy is the primary reason for not getting vaccinated against COVID-19—specifically, concerns over the safety and effectiveness of COVID-19 vaccines.^5^^,^^14^ Our results show that the low adherence to the COVID-19 vaccination is not due to a “hesitancy” about the vaccines, about which people have a favorable opinion, but rather to a “hesitancy” around the perceived threat of the disease. For a large part of the African population, the COVID-19 vaccination seems as unnecessary as vaccination against yellow fever does to people residing in Europe or North America.

In conclusion, our study shows that for both childhood and COVID-19 vaccinations, the rumors around the vaccines are not a significant driver of the insufficient adherence to vaccination. Efforts to promote child vaccination should focus on the organization of the vaccination sessions, friendliness of the health workers, and improving the explanations provided to the parents. Promotion of COVID-19 vaccination should focus on the erroneous ideas about the presence and the toll of the disease in the country, not on the rumors around vaccines, part of the so-called infodemic. Our findings and conclusions, and the communication recommendations based on these, are most likely applicable to all sub-Saharan Africa.

## References

[1] Peckeu-Abboud L , 2022. Drivers of routine and outbreak vaccination uptake in the western Democratic Republic of Congo: an exploratory study in ten health zones. Vaccines (Basel) 10: 1066. 35891230 10.3390/vaccines10071066 PMC9320175

[2] Bobo FT Asante A Woldie M Dawson A Hayen A, 2022. Child vaccination in sub-Saharan Africa: increasing coverage addresses inequalities. Vaccine 40: 141–150. 34794824 10.1016/j.vaccine.2021.11.005

[3] Institut National de la Statistiques and Utica International , 2022. *Enquête nationale sur la fécondité et la mortalité des enfants de moins de cinq ans 2021*. Niamey, Niger: Institut National de la Statistiques; Columbia, MD: Utica International.

[4] Solís Arce JS , 2021. COVID-19 vaccine acceptance and hesitancy in low- and middle-income countries. Nat Med 27: 1385–1394. 34272499 10.1038/s41591-021-01454-y PMC8363502

[5] Faye SLB Krumkamp R Doumbia S , 2022. Factors influencing hesitancy towards adult and child COVID-19 vaccines in rural and urban West Africa: a cross-sectional study. BMJ Open 12: e059138. 10.1136/bmjopen-2021-059138 PMC9013792 35418436

[6] Wonodi C Obi-Jeff C Adewumi F Keluo-Udeke SC Gur-Arie R Krubiner C Jaffe EF Bamiduro T Karron R Faden R, 2022. Conspiracy theories and misinformation about COVID-19 in Nigeria: implications for vaccine demand generation communications. Vaccine 40: 2114–2121. 35153088 10.1016/j.vaccine.2022.02.005 PMC8830779

[7] Seytre B, 2023. Vaccine refusal: a major, underestimated obstacle for the Poliomyelitis Eradication Initiative. Am J Trop Med Hyg 109: 6–9. 37188348 10.4269/ajtmh.23-0154 PMC10323996

[8] Pugliese-Garcia M Heyerdahl LW Mwamba C Nkwemu S Chilengi R Demolis R Guillermet E Sharma A, 2018. Factors influencing vaccine acceptance and hesitancy in three informal settlements in Lusaka, Zambia. Vaccine 36: 5617–5624. 30087047 10.1016/j.vaccine.2018.07.042 PMC6143480

[9] Lewis HC , 2022. SARS-CoV-2 infection in Africa: a systematic review and meta-analysis of standardised seroprevalence studies, from January 2020 to December 2021. BMJ Glob Health 7: e008793. 10.1136/bmjgh-2022-008793 PMC9402450 35998978

[10] Cabore J , 2022. COVID-19 in the 47 countries of the WHO African region: a modelling analysis of past trends and future patterns. Lancet Glob Health 10: e1099–e1114. 35659911 10.1016/S2214-109X(22)00233-9 PMC9159735

[11] World Health Organization , 2022. *Global Excess Deaths Associated with COVID-19*. Available at: https://www.who.int/data/sets/global-excess-deaths-associated-with-covid-19-modelled-estimates. Accessed October 15, 2023.

[12] Wang H , 2022. Estimating excess mortality due to the COVID-19 pandemic: a systematic analysis of COVID-19-related mortality, 2020–21. Lancet 399: 1513–1536. 35279232 10.1016/S0140-6736(21)02796-3 PMC8912932

[13] Seytre B Barros C Bona P Blahima K Rodrigues A Varela O Yoro B Fall B, 2022. Une enquête socio-anthropologique à l’appui de la communication sur le Covid-19 en Afrique de l’Ouest. Med Trop Sante Int 1: MTSIMAGAZINE.N1.2021.106. 10.48327/MTSIMAGAZINE.N1.2021.106 PMC9128470 35686171

[14] Solís Arce JS , 2021. COVID-19 vaccine acceptance and hesitancy in low- and middle-income countries. Nat Med 27: 1385–1394. 34272499 10.1038/s41591-021-01454-y PMC8363502

